# Lupus Nephritis Exhibiting a Membranoproliferative Pattern in Primary Myelofibrosis: A Diagnostic Challenge of Occult SLE

**DOI:** 10.1155/crin/2339466

**Published:** 2026-08-24

**Authors:** Alaa Alem, Raghad Altayyar, Fadyah Alradaddi, Rayan Alahmadi

**Affiliations:** ^1^ Nephrology Section, Department of Medicine, Prince Mohammed Bin Abdulaziz Hospital, National Guard Health Affairs, Madinah, Saudi Arabia, ngha.med.sa; ^2^ Department of Cardiology, Prince Sultan Cardiac Center, Riyadh, Saudi Arabia, pscc.med.sa; ^3^ Department of Internal Medicine, Prince Mohammed Bin Abdulaziz Hospital, National Guard Health Affairs, Madinah, Saudi Arabia, ngha.med.sa

**Keywords:** C1q, JAK2, kidney biopsy, lupus nephritis, membranoproliferative pattern, mycophenolate mofetil, primary myelofibrosis, systemic lupus erythematosus

## Abstract

Lupus nephritis (LN) with a membranoproliferative pattern is an uncommon but recognized renal manifestation of systemic lupus erythematosus (SLE). While autoimmune myelofibrosis (AMF) has been described in association with SLE, the coexistence of primary myelofibrosis (PMF) with this pattern of LN has not been previously documented. We report a 50‐year‐old man with JAK2‐positive PMF, undifferentiated connective tissue disease (UCTD), and Stage 3b chronic kidney disease who developed nephrotic syndrome and worsening kidney function. Initial serologic evaluation revealed positive antinuclear antibody (ANA), low serum complement C3, and negative antidouble‐stranded DNA (anti‐dsDNA) antibodies. Kidney biopsy demonstrated a membranoproliferative pattern of injury with mesangial and subendothelial immune complex deposits staining for IgM and C1q. Subsequent testing revealed high‐titer anticardiolipin IgM antibodies. The patient met the 2019 EULAR/ACR classification criteria for SLE. Induction therapy with high‐dose prednisone and mycophenolate mofetil resulted in a partial remission, with stabilization of renal function on follow‐up. To our knowledge, this is the first reported case of LN with a membranoproliferative pattern of injury in a patient with PMF. The case underscores the diagnostic value of kidney biopsy in uncovering occult systemic autoimmune disease in patients with myeloproliferative neoplasms (MPNs), particularly when the clinical and demographic profile is atypical for SLE.

## 1. Introduction

A membranoproliferative pattern of injury is a histopathological lesion characterized by mesangial hypercellularity, glomerular basement membrane (GBM) thickening, and duplication of capillary walls due to subendothelial immune complex or complement deposition, historically referred to as membranoproliferative glomerulonephritis (MPGN) [1, 2]. Current classification is based on pathogenesis, distinguishing immunoglobulin/immune complex–mediated, complement‐mediated (typically due to alternative pathway dysregulation), and rare forms unrelated to immunoglobulin or complement deposition, such as those caused by thrombotic microangiopathy [3]. Immune complex–mediated cases most commonly arise in the setting of autoimmune diseases, chronic infections, or monoclonal gammopathies [3, 4]. Among autoimmune causes, systemic lupus erythematosus (SLE) is well recognized; although lupus nephritis (LN) typically presents with proliferative lesions (Class III or Class IV under the ISN/RPS classification) [5], a membranoproliferative pattern may occasionally be observed, particularly in chronic or evolving disease. Such cases show mesangial and subendothelial immune complex deposition with classical complement pathway activation. Immunofluorescence (IF) typically demonstrates granular deposition of immunoglobulins and complement components, and strong C1q staining may offer a diagnostic clue even in the absence of a full‐house pattern [6, 7]. This pattern can be diagnostically challenging, particularly when serological findings are initially atypical or incomplete and the patient falls outside the typical SLE demographic profile [8]. In such circumstances, a kidney biopsy often becomes the pivotal diagnostic investigation, providing critical diagnostic guidance even before the systemic autoimmune diagnosis is fully established.

Primary myelofibrosis (PMF) is a clonal myeloproliferative neoplasm (MPN) driven by somatic mutations in JAK2, CALR, or MPL [9]. Although PMF primarily affects hematopoiesis, renal involvement has been reported in a small number of cases, with heterogeneous pathological findings; however, immune complex–mediated glomerular lesions are not typically described in this setting [10, 11]. Beyond their hematological manifestations, clonal MPNs such as PMF are increasingly recognized as chronic inflammatory disorders characterized by sustained cytokine activation and altered immune homeostasis, contributing to systemic immune dysregulation [12]. This evolving understanding provides a conceptual framework for the potential coexistence of clonal myeloid neoplasia and overt autoimmune disease.

Reported cases of myelofibrosis in SLE relate predominantly to autoimmune myelofibrosis (AMF), a nonclonal, immune‐mediated entity pathophysiologically distinct from PMF [13–15]. The coexistence of SLE‐related glomerular disease with clonal PMF, by contrast, has not been previously documented.

We report the case of a 50‐year‐old man with JAK2‐positive PMF who developed nephrotic syndrome and was ultimately diagnosed with LN with a membranoproliferative pattern of injury.

## 2. Case Presentation

A 50‐year‐old male was referred to the nephrology clinic for evaluation of progressive renal dysfunction. His medical history was notable for JAK2‐positive PMF, undifferentiated connective tissue disease (UCTD) manifested as polyarthralgia and previously controlled on hydroxychloroquine, hypertension, gout, benign prostatic hyperplasia (BPH), and a remote ischemic cerebrovascular accident (CVA) in his 20s. Chronic kidney disease (CKD) Stage 3b had been recognized two years earlier, with serum creatinine levels ranging from 150 to 170 μmol/L. His regular medications at presentation included ruxolitinib, hydroxychloroquine, allopurinol, amlodipine, and a combination of dutasteride/tamsulosin.

The patient described a 1‐year history of gradually worsening bilateral lower limb edema extending up to the knees, frothy urine, and increased urinary frequency. He denied hematuria, dysuria, fever, weight loss, night sweats, jaundice, abdominal distension, shortness of breath, or chest pain. He reported no photosensitivity, oral ulcers, skin rash, or joint pain. He reported no recent infections or exposure to nephrotoxic medications, and there was no family history of autoimmune or renal diseases.

On examination, the patient was obese (body mass index 36 kg/m^2^) with a blood pressure of 140/90 mmHg. Bilateral pitting edema extended to the knees, without erythema or signs of cellulitis. There was no lymphadenopathy, malar rash, oral ulcer, alopecia, or active synovitis. Abdominal examination revealed hepatosplenomegaly without ascites. Cardiovascular and respiratory examinations were unremarkable.

The complete blood count showed leukocytosis (WBC 27.0 × 10^9^/L), thrombocytopenia (platelet count 112 × 10^9^/L), and anemia (hemoglobin 11 g/dL). Renal function had declined from baseline, with serum creatinine of 220 μmol/L and an estimated glomerular filtration rate (eGFR) of 29 mL/min/1.73 m^2^. Serum albumin was reduced to 29 g/L, and the lipid profile showed dyslipidemia (LDL‐cholesterol 3.04 mmol/L). Liver function tests were normal. Urine analysis demonstrated 3+ proteinuria without hematuria, leukocyte esterase, or pyuria. A 24‐h urine collection revealed proteinuria of 17.86 g/day.

Initial immunological work‐up revealed a positive antinuclear antibody (ANA), reduced complement component C3 with a normal C4, and negative antidouble‐stranded DNA (anti‐dsDNA) antibodies. Additional autoimmune serology, including anti‐SSA, anti‐SSB, rheumatoid factor (RF), and anticyclic citrullinated peptide (anti‐CCP) antibodies, was negative. Following kidney biopsy, additional testing revealed anticardiolipin IgM antibodies at high titer (127 MPL units); anticardiolipin IgG and IgA antibodies, lupus anticoagulant, and Anti‐β2‐glycoprotein I antibodies were negative. Perinuclear antineutrophil cytoplasmic antibodies (p‐ANCAs), cytoplasmic ANCAs (c‐ANCAs), and cryoglobulins were negative. Serum protein electrophoresis with immunofixation was unremarkable. Bence Jones protein was absent, and the serum free kappa/lambda light‐chain ratio was within normal limits. Serologies for hepatitis B, hepatitis C, and HIV were negative.

Renal ultrasound demonstrated kidneys of preserved size with bilaterally mildly increased parenchymal echogenicity and preserved corticomedullary differentiation. There was no evidence of hydronephrosis or structural abnormalities. Doppler ultrasound showed normal arterial and venous flow with no evidence of vascular compromise.

**FIGURE 1:**
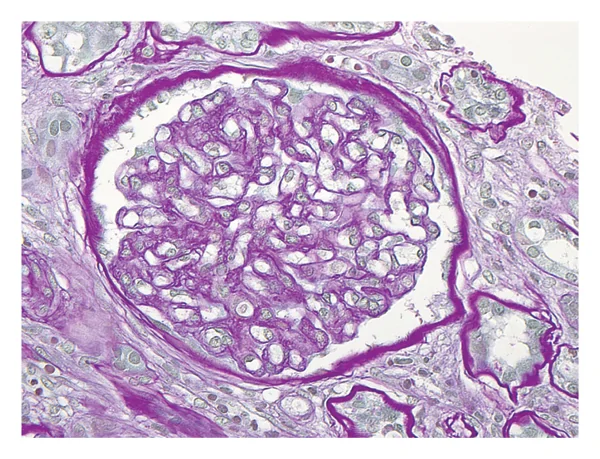
PAS‐stained slide showing glomerulus with mesangial hypercellularity and GBM thickening with duplication of the basement membrane (membranoproliferative pattern of injury).

**FIGURE 2:**
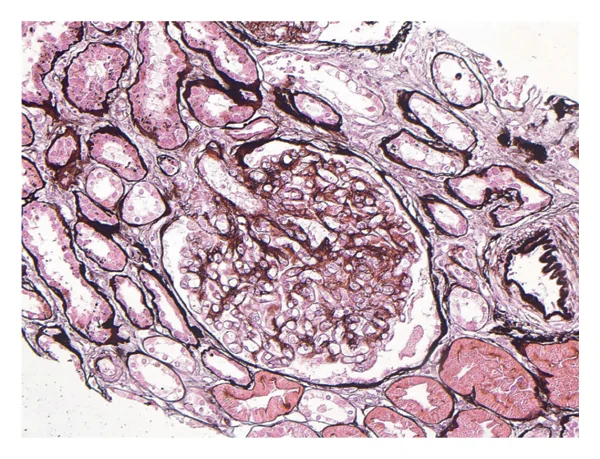
Jones‐stained slide showing GBM thickening and segmental duplication of the basement membrane.

**FIGURE 3:**
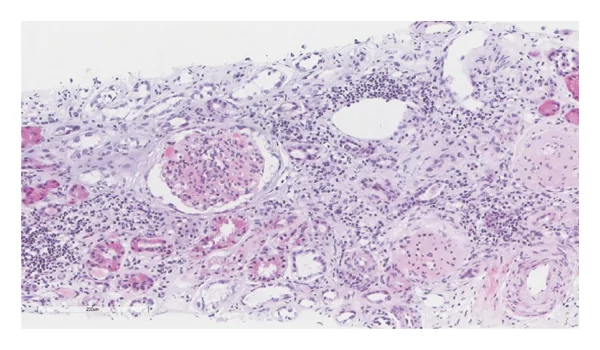
Low‐power H&E‐stained slide showing chronic changes with global glomerular scarring (blue arrows) and severe IFTA. One glomerulus shows mesangial expansion and hypercellularity (red arrow).

**FIGURE 4:**
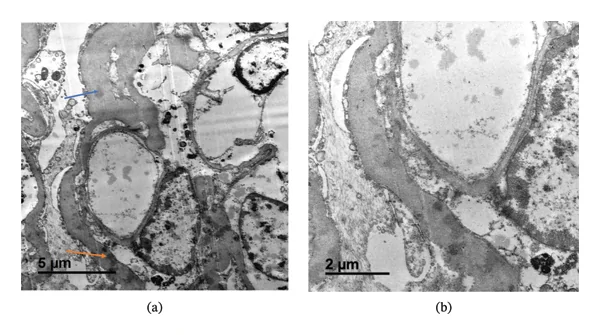
Electron microscopy images. (a) Lower‐power view (scale bar 5 μm) showing mesangial (blue arrow) and subendothelial electron‐dense deposits (orange arrow) with multilayering of the GBM. (b) Higher‐magnification view (scale bar 2 μm) showing GBM splitting and scattered subendothelial electron‐dense deposits (blue arrow).

A percutaneous kidney biopsy was performed. On light microscopy, the specimen contained 16 glomeruli, of which 4 were globally sclerosed, and 2 exhibited features of focal segmental glomerulosclerosis (FSGS). The remaining glomeruli demonstrated diffuse mesangial hypercellularity and GBM thickening with double contours, consistent with a membranoproliferative pattern of injury. No spikes or craters were identified. The tubulointerstitial compartment showed advanced chronic damage, with interstitial fibrosis and tubular atrophy (IFTA) involving approximately 60%–65% of the cortex. Vascular changes consisted of arteriolar hyalinosis and mild arteriolosclerosis. Congo red staining for amyloid was negative. IF on frozen sections demonstrated granular mesangial and capillary wall staining for IgM (2+), C1q (1–2+), and weak IgA (0–1+), with no IgG staining. Staining for C3 and C4 was weak and considered nonspecific. Both kappa and lambda light chains were weakly positive within tubular casts, with no glomerular light chain restriction. Linear albumin staining was weak, and fibrinogen staining was noncontributory. On electron microscopy, mesangial and subendothelial electron‐dense immune‐type deposits were identified, with mesangial cell interposition and new basement membrane formation accounting for the double contours observed on light microscopy. No subepithelial or intramembranous deposits, organized substructures (such as microtubules or fingerprints), or tubuloreticular inclusions were identified. The combined light microscopic, IF, and ultrastructural findings were considered most compatible with proliferative LN exhibiting a membranoproliferative pattern of injury. Representative histological findings are shown in Figures 1, 2, 3, and 4. A summary of IF findings is presented in Table 1.

**TABLE 1:** Summary of immunofluorescence staining results.

Marker	Intensity	Pattern/location
IgG	Negative	—
IgA	0–1+	Granular, mesangial, and capillary wall
IgM	2+	Granular, mesangial, and capillary wall
C3	Weak, nonspecific	—
C4	Weak, nonspecific	—
C1q	1–2+	Granular, mesangial, and capillary wall
Kappa light chain	Weak	Polytypic, in tubular casts (no glomerular restriction)
Lambda light chain	Weak	Polytypic, in tubular casts (no glomerular restriction)
Albumin	Weak	Linear, along GBM
Fibrinogen	Noncontributory	—

### 2.1. Diagnosis

Integration of the clinical, serological, and pathological findings supported a final diagnosis of LN with a membranoproliferative pattern of injury, reflecting newly recognized SLE in a patient with established JAK2‐positive PMF.

Although the IF pattern lacked some classical features of LN, specifically the absence of IgG, the absence of a “full‐house” pattern, and the absence of tubuloreticular inclusions on electron microscopy, the combination of prominent C1q deposition, mesangial and subendothelial immune‐complex deposits, hypocomplementemia, ANA positivity, and high‐titer anticardiolipin IgM positivity together supported an immune complex–mediated lupus etiology. The patient met the 2019 EULAR/ACR classification criteria for SLE: ANA positivity satisfied the entry criterion, and the additive weighted score reached ≥ 10 points, contributed by the renal biopsy findings interpreted as showing proliferative LN morphology (10 points), low complement C3 (3 points), and anticardiolipin IgM positivity at high titer (2 points).

Principal alternative etiologies of an immune complex–mediated membranoproliferative pattern were systematically excluded. The renal lesion was therefore interpreted as an LN variant rather than an MPGN from an alternative cause. C3 glomerulopathy was considered unlikely in the absence of C3‐dominant IF. Monoclonal gammopathy was excluded by negative serum immunofixation, a normal serum‐free light‐chain ratio, and polytypic light‐chain staining on biopsy. Infection‐related immune complex glomerulonephritis was excluded by negative hepatitis B, hepatitis C, and HIV serologies, together with the absence of clinical or laboratory evidence of active infection. A PMF‐attributable glomerular lesion and drug‐induced lupus (DIL) secondary to therapeutic agents used for PMF were also considered but judged unlikely on clinical, serological, and histological grounds.

### 2.2. Management and Clinical Course

**FIGURE 5:**
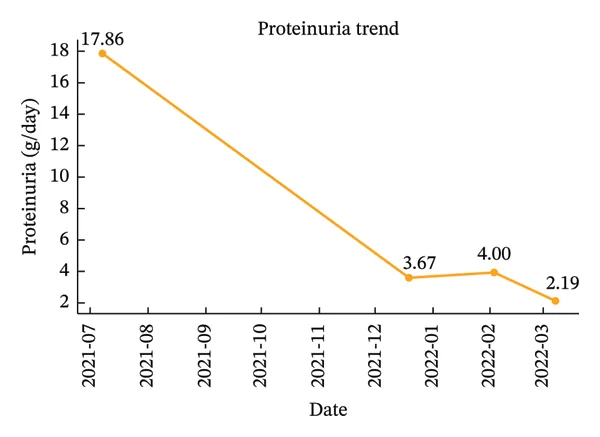
Trend of proteinuria in response to treatment.

The patient was initially treated with oral diuretics and a renin–angiotensin system inhibitor for volume control and antiproteinuric effect. Despite conservative therapy, proteinuria remained in the nephrotic range, with urine protein‐to‐creatinine ratio measurements indicating proteinuria of approximately 20 g/g equivalent. Following histopathological confirmation of the diagnosis, induction therapy was commenced with oral prednisone 60 mg daily, with a planned taper. After several weeks, mycophenolate mofetil (MMF) was added at induction dosing of 3 g/day, with the staged introduction reflecting the patient’s underlying PMF and baseline thrombocytopenia. Hydroxychloroquine was continued throughout as an adjunctive immunomodulator. The induction regimen was well tolerated. 24‐h proteinuria decreased from 17.86 g/day at baseline to 2.19 g/day following the introduction of immunosuppression, with sustained reduction over subsequent follow‐up, consistent with a partial remission. Renal function remained stable throughout follow‐up. Prednisone was tapered to a maintenance dose of 5 mg daily, and MMF was continued as maintenance therapy. The trend in proteinuria during induction and maintenance is illustrated in Figure 5.

## 3. Discussion

This case illustrates a rare and diagnostically challenging overlap between LN with a membranoproliferative pattern of injury and PMF. Although each of these conditions is well described individually, their coexistence in a single patient has not, to our knowledge, been previously described. The patient had a preexisting diagnosis of UCTD controlled on hydroxychloroquine. At presentation, autoimmune evaluation revealed a positive ANA and reduced complement C3 with a normal C4 and negative anti‐dsDNA antibodies. These findings were suggestive of an autoimmune process but were not specific enough to confirm SLE. The kidney biopsy was the pivotal diagnostic event, demonstrating a membranoproliferative pattern with prominent C1q deposition that, together with subsequent high‐titer anticardiolipin IgM antibodies, supported the diagnosis of LN as the renal manifestation of SLE evolving from the patient’s preexisting UCTD.

A membranoproliferative pattern represents a histological pattern of glomerular injury characterized by mesangial hypercellularity, GBM thickening, and duplication of capillary walls due to subendothelial immune complex or complement deposition [1, 2]. The current pathogenesis‐based classification categorizes this pattern into immunoglobulin/immune complex–mediated, complement‐mediated, and forms unrelated to immunoglobulin or complement deposition, such as those caused by thrombotic microangiopathy [3]. In this patient, kidney biopsy demonstrated mesangial and subendothelial immune complex deposition with granular staining for IgM and C1q, consistent with an immune complex–mediated process. Complement‐mediated disease was unlikely given the absence of C3‐dominant staining. In addition, monoclonal immunoglobulin–associated disease was excluded by negative serum and urine electrophoresis and a normal free light chain ratio. Infectious etiologies, including hepatitis B, hepatitis C, and HIV, were also excluded. Taken together, these findings support an immune complex–mediated process, which in the appropriate clinical and serologic context is most consistent with LN presenting with a membranoproliferative pattern of injury.

While full‐house IF is considered typical for LN, its absence does not preclude the diagnosis. In such cases, strong C1q staining remains a reliable histopathological clue to lupus‐related glomerular disease, particularly when supported by serological findings such as hypocomplementemia and ANA positivity [6, 7]. In our patient, the absence of full‐house IF was initially atypical for LN; only IgM and C1q staining were present. However, the prominent C1q deposition, combined with the serological profile, was consistent with the diagnosis. The patient’s demographic background also contributed to the initial uncertainty, as SLE predominantly affects women of reproductive age, with a female‐to‐male ratio of approximately 9:1 [8], and is uncommon in middle‐aged males. Our 50‐year‐old male patient sits outside this typical population. This case illustrates that LN can occur with neither full‐house IF nor classical clinical demographics and underscores the value of integrating histopathological findings with serological evaluation when individual features are atypical.

The association between SLE and myelofibrosis is recognized in the literature, although it is uncommon and remains incompletely understood. Most reported cases involve AMF, a nonclonal, immune‐mediated bone marrow process associated with systemic autoimmune diseases. Gutarra‐Arana et al. reported a case of AMF associated with SLE, in which pancytopenia improved after high‐dose corticosteroids and intravenous immunoglobulin, although recurrent cytopenic episodes occurred during the disease course [15]. Mbonu et al. described a case in which AMF was the presenting clinical feature of underlying SLE, suggesting that bone marrow fibrosis may occasionally represent an early or atypical autoimmune manifestation [13]. Similarly, Español et al. presented a pediatric case of co‐occurring SLE, LN, and AMF in the setting of monosomy 7 [14]. In contrast, our patient was diagnosed with PMF, a clonal MPN caused by somatic mutations in JAK2, CALR, or MPL [9] and pathophysiologically different from AMF.

Although PMF primarily affects hematopoiesis, renal involvement in MPNs is uncommon and heterogeneous, more frequently manifesting as a distinctive MPN‐related glomerulopathy with mesangial proliferative and sclerosing features, FSGS, thrombotic microangiopathy, and chronic tubulointerstitial or vascular changes [10, 11]. Several mechanistic pathways have been proposed by which PMF could contribute to renal involvement. PMF is a chronic inflammation–driven neoplasm in which constitutive JAK‐STAT activation sustains the release of proinflammatory cytokines, including IL‐1β, IL‐6, IL‐8, TNF‐α, and TGF‐β, with associated immune dysregulation [12]; these mediators have been associated with endothelial injury and renal microvascular damage and with the development of autoantibodies and altered T‐cell function. In addition, paraneoplastic glomerulonephritis associated with hematological malignancies shows distinct lineage‐specific patterns: among myeloid neoplasms, mesangioproliferative glomerulonephritis and FSGS predominate, whereas membranoproliferative and membranous lesions with prominent immune complex deposition are characteristic of B‐cell lymphoid disorders and are typically driven by monoclonal immunoglobulin or cryoglobulin [16]. None of these mechanisms or patterns readily accounts for the membranoproliferative pattern with prominent C1q deposition and supporting autoimmune serological profile observed in our patient. The absence of a detectable monoclonal protein, the polytypic nature of the immune complex deposition, the autoimmune serology, and the sustained renal response to lupus‐directed immunosuppression without modification of PMF treatment together favor LN with a membranoproliferative pattern of injury rather than a PMF‐attributable lesion. Nevertheless, a contributory role of the PMF inflammatory environment in promoting immune dysregulation cannot be entirely excluded.

Another consideration in this case is whether the development of SLE could represent DIL, particularly in the context of pharmacological treatment for PMF. DIL typically presents with milder systemic manifestations compared to idiopathic SLE, although renal involvement has been reported, albeit infrequently. These atypical renal manifestations include immune complex–mediated glomerular injury with a membranoproliferative pattern, which has been described in rare cases, indicating that immune complex deposition may occur in selected drug‐induced settings [17–19]. Commonly implicated agents include hydralazine, procainamide, and anti‐TNFα therapies, with additional associations reported for interferon‐α, isoniazid, and minocycline [17, 18, 20]. Ruxolitinib, the JAK1/2 inhibitor used in this patient for PMF, has not been clearly associated with DIL in the published literature. To our knowledge, lupus‐like syndromes and autoimmune adverse events have not been reported in major clinical studies of ruxolitinib, including the pivotal COMFORT‐I trial of myelofibrosis [21]. Furthermore, ruxolitinib was not among the 118 drugs identified in a comprehensive analysis of the WHO global pharmacovigilance database for DIL [22]. In our patient, a comprehensive review of both past and current medications revealed no exposure to drugs classically associated with DIL. Although antihistone antibody testing was not performed, which limits complete exclusion of DIL, the overall serological profile—positive ANA and high‐titer anticardiolipin IgM antibodies, low complement C3, and absence of anti‐dsDNA antibodies—together with prominent C1q deposition on kidney biopsy supports a diagnosis of idiopathic SLE. Notably, the patient had carried an established diagnosis of UCTD with polyarthralgia, controlled on hydroxychloroquine, well before the diagnosis of PMF and the initiation of any PMF‐directed therapy. This indicates that autoimmune disease predated any drug exposure that could have triggered it. Therefore, while the potential for DIL with renal involvement remains an important diagnostic consideration, it appears unlikely in our case.

The management of LN with a membranoproliferative pattern follows established treatment principles for proliferative LN. Induction therapy typically includes high‐dose corticosteroids in combination with immunosuppressive agents such as MMF or cyclophosphamide. MMF has demonstrated comparable efficacy to cyclophosphamide in inducing remission and preserving long‐term renal function, with a more favorable safety profile [23]. The 2021 KDIGO clinical practice guideline, which was the prevailing standard at the time of management, endorses MMF combined with corticosteroids as a first‐line treatment for proliferative LN [24]. In our patient, induction therapy with high‐dose oral prednisone followed by the addition of MMF resulted in a partial remission, proteinuria decreased from a peak of 17.86 g/day to 2.19 g/day at the last follow‐up, and renal function remained stable. The substantial chronic damage demonstrated on biopsy (IFTA involving 60%–65% of the cortex) is a recognized predictor of partial rather than complete remission in LN and likely contributed to the response observed. Following the partial remission, the patient was transitioned to maintenance therapy with low‐dose prednisone (5 mg daily) and MMF.

### 3.1. Conclusion

This case highlights the critical diagnostic role of kidney biopsy in patients with MPNs presenting with otherwise unexplained proteinuria and renal impairment. The coexistence of biopsy‐proven LN with a membranoproliferative pattern of injury and PMF has not, to our knowledge, been previously described. In this patient, the kidney biopsy was the pivotal event that integrated previously nonspecific serological findings into a coherent diagnosis of SLE evolving from previously diagnosed UCTD. Disease‐directed immunosuppression with corticosteroids and MMF resulted in a partial remission, with the substantial chronic damage demonstrated on biopsy likely contributing to the incomplete remission observed. Clinicians should maintain a high index of suspicion for underlying autoimmune disease in patients with MPNs who present with otherwise unexplained renal abnormalities and should not defer kidney biopsy even when the clinical and demographic profile is atypical for SLE.

**Nomenclature**
AMF: Autoimmune myelofibrosis
ANA: Antinuclear antibody
Anti‐dsDNA: Anti‐double‐stranded DNA
BPH: Benign prostatic hypertrophy
CKD: Chronic kidney disease
CVA: Cerebrovascular accident
DIL: Drug‐induced lupus
eGFR: Estimated glomerular filtration rate
FSGS: Focal segmental glomerulosclerosis
GBM: Glomerular basement membrane
IF: Immunofluorescence
IFTA: Interstitial fibrosis and tubular atrophy
LN: Lupus nephritis
MMF: Mycophenolate mofetil
MPGN: Membranoproliferative glomerulonephritis
MPN(s): Myeloproliferative neoplasm(s)
PMF: Primary myelofibrosis
SLE: Systemic lupus erythematosus
UCTD: Undifferentiated connective tissue disease

## Author Contributions

Alaa Alem: conceptualization, supervision, literature review, writing–final draft, and writing–review and editing. Raghad Altayyar: case documentation, literature review, and writing–original draft. Fadyah Alradaddi: clinical data acquisition, figure preparation, and writing–original draft. Rayan Alahmadi: clinical data acquisition and writing–original draft.

## Funding

No funding was received for this research.

## Ethics Statement

Ethical approval is waived by a local ethics committee.

## Consent

Written informed consent for publication was obtained from the patient’s legal representative, his wife, as the patient had passed away.

## Conflicts of Interest

The authors declare no conflicts of interest.

## Data Availability Statement

Data sharing is not applicable to this article as no datasets were generated or analyzed during the current study.

## Supporting Information

Additional supporting information can be found online in the Supporting Information section.

## Supporting information


**Supporting Information** CARE checklist of information to include when writing a case report.
